# Autoimmune Rhombencephalitis as a Presentation of Post-COVID-19 Syndrome: A Case Report

**DOI:** 10.7759/cureus.79517

**Published:** 2025-02-23

**Authors:** Alaa S Mohamed, Neesha Jahani, Mingyu Li, Manan Shah, Askiel Bruno, Dilip Singh

**Affiliations:** 1 Neurology, Augusta University, Augusta, USA; 2 Neurology, Augusta University Medical College of Georgia, Augusta, USA

**Keywords:** autoimmune, brain stem, cerebellum, covid 19, post-covid-19, rhombencephalitis, sars-cov

## Abstract

There has been a multitude of neurological sequelae associated with the 2019 SARS-CoV-2 outbreak. We describe a unique case of a 31-year-old individual who developed post-infectious autoimmune rhombencephalitis approximately two weeks after testing positive for coronavirus disease 2019 (COVID-19). Treatment with high-dose corticosteroids with four weeks of taper resulted in excellent clinical recovery without any relapse. Thus, our findings support the use of immunotherapy as a potential treatment in cases of post-COVID-19 autoimmune rhombencephalitis. Furthermore, this case highlights the importance of considering post-infectious COVID-19 sequelae as a differential diagnosis in patients presenting with rhombencephalitis in the appropriate clinical setting.

## Introduction

With over six million fatalities worldwide, the ongoing SARS-CoV-2 outbreak has proven to be detrimental (World Health Organization, 2022). Several studies have shown that acute respiratory distress is the most common complication associated with coronavirus disease 2019 (COVID-19). However, there has been a growing number of reports of severe neurological manifestations, such as strokes, encephalitis, and seizures, in infected patients [1]. Several mechanisms have been proposed for how the virus infects the central nervous system (CNS) including direct viral invasion of the nervous system, neurologic injury from systemic dysfunction, and parainfectious or postinfectious autoimmune attack leading to neurological manifestations [2]. We describe a patient with brainstem and cerebellar lesions consistent with rhombencephalitis (RE) following COVID-19, a rare complication with only a few reported cases in the literature.

## Case presentation

A 31-year-old Caucasian male with a past medical history significant for recent COVID-19 infection, current tobacco and marijuana use, and former alcohol abuse presented to a community hospital with a new onset of right-sided weakness, right facial droop, slurred speech, fever, and altered mental status. The condition started five days prior with a severe occipital headache, fever, and persistent hiccups and progressed to unilateral weakness and dysarthria. The patient tested positive for COVID-19 two weeks before the onset of symptoms, which manifested as a mild flu-like illness. He did not require any treatment for this. The patient reported being vaccinated for COVID-19.

At the referring hospital, the patient was initially evaluated for a stroke with a negative workup barring a brain MRI that reportedly revealed an increased T2 signal in the pons, cerebral peduncle, and cerebellar peduncle. Because of the community hospital's limited resources and the clinical condition's urgency, the patient was transferred to our institution to optimize care.

**Figure 1:**
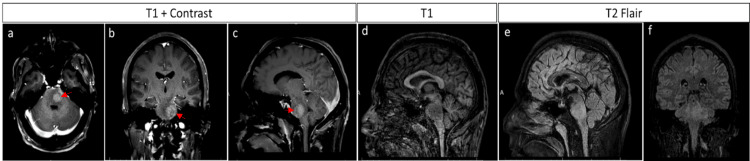
MRI Brain with contrast on the day of admission showing a heterogenous lesion on the left pons surrounded by vasogenic edema. The lesion is heterogeneously enhancing with contrast (a) + (b) + (c) (red arrow). The lesion appears hypointense in T1 (d) and hyperintense on T2 (e) + (f). FLAIR: Fluid-Attenuated Inversion Recovery.

Upon arrival, the patient was febrile with a temperature of 39 degrees Celsius. Initial exam findings revealed an alert and oriented patient with moderate dysarthria, right lower facial weakness, and ophthalmoplegia of the left eye. Additionally, the patient had numbness and weakness (Medical Research Council grade 3) on the right upper and lower extremities. A brain MRI with and without contrast was repeated and confirmed a brainstem lesion centered in the left pons with mild enhancement surrounded by moderate vasogenic edema (Figure 1). Diagnostic workup, including infectious, autoimmune, and cytology studies from both serum and cerebrospinal fluid (CSF), was negative, as illustrated in Table 1.

**Table 1:** Diagnostic workup CSF: Cerebrospinal Fluid, HSV-1/HSV-2: Herpes Simplex Virus types 1 and 2, VZV: Varicella Zoster Virus, EBV: Epstein-Barr Virus , LCMV: Lymphocytic Choriomeningitis Virus, PCR: Polymerase Chain Reaction, AFB: Acid-Fast Bacilli, ANA: Antinuclear Antibody , DS DNA: Double-Stranded DNA, Scl 70: Scleroderma-70 (Topoisomerase I), SSA: Sjögren's Syndrome Antibody ,U1RNP: U1 Small Nuclear Ribonucleoprotein, Anti-Ma/Ta: Anti-Ma and Anti-Ta Antibodies (paraneoplastic antibodies), Anti-MOG: Myelin Oligodendrocyte Glycoprotein Antibody, Anti-GM1: Anti-Ganglioside GM1 Antibody, Anti-GQ1b: Anti-Ganglioside GQ1b Antibody, Anti-AQP-4: Aquaporin-4 Antibody, RBCs: Red Blood Cells, IgG: Immunoglobulin G.

Test	Result	Reference range
Lumbar Puncture Findings		
- Fluid Appearance	Clear	Clear
- Opening Pressure	14	10-20 cm H₂O
- Glucose	61	40-70 mg/dL
- Total Nucleated Cells	184	0-5 cells/µL
- RBCs	2	0-5 cells/µL
- Neutrophils	65	0-6% of total nucleated cells
- Lymphocytes	30	30-80% of total nucleated cells
- Monocytes/Macrophages	5	0-10% of total nucleated cells
- Protein Levels	Mildly elevated (59 mg/dL)	15-45 mg/dL
Infectious Workup CSF and/or Serum		
HSV-1/HSV-2	Negative	
VZV	Negative	
EBV	Negative	
Enterovirus	Negative	
LCMV	Negative	
Histoplasma Antigen/Antibody	Negative	
Listeria PCR	Negative	
West Nile Virus	Negative	
Eastern Equine Virus	Negative	
AFB Antibodies	Negative	
Vasculitis/Autoimmune Workup		
ANA	Negative	
DS DNA	Negative	
Scl 70	Negative	
Smith	Negative	
SSA	Negative	
U1RNP	Negative	
Anti-Ma/Ta	Negative	
Anti-MOG	Negative	
Anti-GM1	Negative	
Anti-GQ1b	Negative	
Anti-AQP-4	Negative	
Other CSF Tests		
Flow Cytometry	Negative for monoclonal B or T cells	
Cytology	Negative for malignant cells	
SARS-CoV-2 Testing		
Nasopharyngeal PCR	Negative on presentation; Positive 2 weeks prior	
IgG	Positive (indicating past infection and/or previous vaccination)	

Given the patient’s occupational background of handling raw chicken meat, he was started on IV vancomycin, ceftriaxone, and ampicillin while the Listeria polymerase chain reaction (PCR), CSF cultures, and blood cultures were pending. On the second day, the patient continued to worsen acutely, with worsening of the bulbar symptoms, inability to swallow, and progressive weakness. Hence, he was started on a five-day course of IV methylprednisolone 1 gm daily empirically for suspicion of a malignant/inflammatory process along with continuing the antibiotics. Physical examination findings at this time were significant for severe dysarthria, cranial nerves IV, VI, and XII dysfunction, impaired strength bilaterally (right>left), decreased sensation to light touch on the right side, and a positive right-foot Babinski reflex.

**Figure 2:**
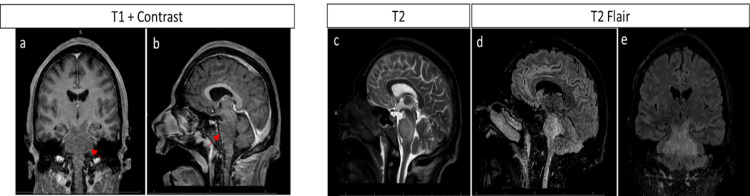
MRI Brain with contrast on day four of receiving steroids showing expansion of the lesion to bilateral pons, cerebellar peduncles and touching mildly the cerebellum. Notice that the enhancement of the lesion and the edema had faded after receiving steroids (a) +(b) (red arrow). (c), (d) and (e) are showing the expanded lesion on T2 and T2 FLAIR imaging. FLAIR: Fluid-Attenuated Inversion Recovery.

Five days after initial admission, the patient underwent emergent intubation for impending respiratory failure as a result of increased secretions, hypoxemia, and decreased mental status, the patient was intubated for airway protection. A repeat lumbar puncture (LP) was performed resulting in an opening pressure of 16. Fluid analysis showed a colorless fluid, absent xanthochromia, and improving lymphocytic pleocytosis (42 total nucleated cells, 4 RBCs, 0 neutrophils, 96 lymphocytes, and 4 monocytes/macrophages). Glucose and protein levels remained unchanged (protein level at 59). A repeat MRI of the brain, with and without contrast, was performed after four doses of methylprednisolone and six days following the first MRI. It revealed involvement of the right pons and cerebral peduncle (Figure 2). Imaging of the spine was also obtained but was unremarkable. Antibiotic therapy was stopped as most of the infectious workup was negative. The rest of the auto-immune workup remained negative. CSF cytology and flow cytometry were negative for abnormal cells. At this point, the patient was ultimately diagnosed with post-infectious rhombencephalitis secondary to his recent SARS-CoV-2 infection after the exclusion of other potential etiologies. 

On the eighth day of hospitalization, the patient started to show mild improvement after the completion of five days of IV methylprednisolone 1 gm daily. Continued improvement without antibiotic therapy and with steroids pointed towards the auto-immune nature of the disease process. Then the patient was put on three weeks of steroid taper starting with oral prednisone 80 mg. On day 12, the patient continued to improve, and he was discharged home per his request, despite our recommendation for needing a rehabilitation program. He was still ataxic bilaterally with notable weakness on the right side on discharge. After four weeks, the patient was seen in the clinic after being off of steroids for one week. He reported minimal symptoms in the form of occasional headaches and irritability. His neurologic exam was normal except for brisk deep tendon reflexes in all four limbs.

## Discussion

COVID-19 has been linked to a variety of neurological sequelae such as cerebrovascular diseases and encephalitis as well as neuroimmunological manifestations. Some of these complications are thought to be caused concurrently with acute illness; however, most seem to present after recovery or post-vaccination [1]. A suggested mechanism for the neurological complications of COVID-19 is direct SARS-CoV-2 invasion of neural cells through angiotensin-converting enzyme two receptors [3], but the mechanism of autoimmune neurological sequelae associated with COVID-19 remains uncertain. For example, an autopsy performed on COVID-19 patients found the brainstem to be directly invaded by the virus and was thought to be a contributing factor to respiratory failure by specifically affecting the respiratory center in the medulla oblongata [4]. A review of current literature has only limited cases outlining RE as an outcome after SARS-CoV-2 infection. Here we present a case of RE that occurred two weeks after COVID-19 infection.

Rhombencephalitis is a rare condition that results in a wide variety of neurological symptoms due to inflammation of the brainstem and cerebellum. The most common etiologies include infections, autoimmune diseases, and paraneoplastic syndromes. Of the three categories, infectious etiology strikes as the most common cause of rhombencephalitis with the most well-known culprits being Listeria monocytogenes, Enterovirus 71, and herpes simplex virus (HSV) [1]. Among the autoimmune etiologies, Behcet’s disease is the most common [5], but RE has also been found to be associated with Guillain-Barre syndrome (GBS), acute disseminated encephalomyelitis (ADEM), Bickerstaff brainstem encephalitis, and transverse myelitis. Other acute demyelinating diseases like neuromyelitis optica (NMO), multiple sclerosis (MS), and myelin oligodendrocyte glycoprotein (MOG)-related disorders have also been reported as a cause. All these conditions were considered as part of the differentials in our case.

Beginning with the infectious etiologies in our case, Listeria was high on our differential considering the patient’s occupational background plus the borderline white blood cell count in his CSF. Our patient’s clinical history even paralleled the typical biphasic course of a Listeria CNS infection starting with a headache, fever and chills then progressing to ataxia, CN III palsy, altered mental status, and hemiparesis [6]. Yet, both the PCR, CSF cultures, and blood cultures were negative along with a minimal response to antibiotics effectively making Listeria less likely as the cause. Similarly, lack of a clinical picture and negative lab results made other viral infectious agents less likely in our case.

Autoimmune disorders, including Bickerstaff’s, ADEM, NMO, and MS, were considered but deemed less likely due to atypical clinical and radiological features, as well as negative lab findings (anti-GM1, anti-GQ1, anti-MOG, anti-AQP-4, and oligoclonal bands). Neuro-Behcet was ruled out as the patient had no history of orogenital ulcers. In addition, a concern for neoplasm, specifically glioma, was initially suspected due to radiological concerns, the acute clinical presentation and recovery, along with negative CSF cytology and flow cytometry, made this diagnosis unlikely. Given the lack of supporting labs and historical clues, post-COVID-19 exposure emerged as the most likely diagnosis by exclusion.

Regarding the radiological finding, our patient showed brain enhancing lesion in the left pons that evolved abruptly to include bilateral lower brainstem and cerebellar peduncles and cerebellum white matter. The enhancement seems to be rapidly resolved after steroid administration (Figure 2). Interestingly the lesion was surrounded by vasogenic edema. Findings were concerning for brain mass. However, the clinical picture, acute rapid progressive onset, unremarkable labs, and rapid improvement to steroids made the diagnosis less likely. The lesion didn’t show restriction diffusion, compared to some cases in the literature [7,8].

According to the few cases that are reported for COVID-related RE, most of the cases had significant improvement and dramatic response to steroids only. However, few of them needed further courses of plasmapheresis and intravenous immunoglobulin (IVIG) [7,9]. Our case had almost returned to his baseline with steroids only followed by a prolonged taper. Our patient started to improve to steroids on the fourth day of starting steroids. Delayed improvement to steroids was thought to be affected by the patient’s poor general condition on the exam due to recent intubation. A case reported by Wood et al. revealed that their patient significantly deteriorated after stopping steroids abruptly without weaning, which was deemed a repeat of another steroid course with slow tapering [8]. Moreover, isolated RE was also reported after COVID vaccination [10]. Reviewing the literature, the duration before developing RE ranged from one week and up to one month post-SARS [7].

## Conclusions

From a mild cough and headache to severe respiratory distress and demise, it is well-known that SARS-CoV-2 can cause a large array of both respiratory and neurological symptoms. What is more obscure is the severity of the post-infectious neurological sequelae. Our case adds to the literature by describing isolated neurological manifestations after recovery from a minor SARS-CoV-2 infection with excellent response to pulse dose of steroids. We believe that an earlier start of immunotherapy would prevent serious complications of the disease and may lead to excellent neurologic recovery. 

We believe this case to be a good example of a typical presentation of post-COVID exposure rhombencephalitis which has poor representation in current literature. Our case highlights the importance of considering RE in patients who have been recently exposed to COVID-19 and present with bulbar and cerebellar symptoms. 
